# Management of neurogenic bladder in patients with spinal cord injuries/disorders and end stage renal disease: a case series

**DOI:** 10.1038/s41394-024-00623-8

**Published:** 2024-03-04

**Authors:** Rajbir Chaggar, Lance L. Goetz, Jordan Adler, Mohammed B. A. Bhuiyan, Sean McAvoy, Jeffrey Tubbs

**Affiliations:** 1 Virginia Commonwealth University Health System, Richmond, VA USA; 2 Central Virginia Veterans Affairs Health Care System, Richmond, VA USA; 3 Syracuse VA Medical Center, Syracuse, NY USA

**Keywords:** Neurogenic bladder, Spinal cord diseases, Rehabilitation, End-stage renal disease

## Abstract

**Introduction:**

Patients with spinal cord injuries/disorders (SCI/D) often suffer from bladder dysfunction, commonly referred to as neurogenic bladder or neurogenic lower urinary tract dysfunction (NLUTD). Standard urologic evaluation and management help to minimize complications such as vesicoureteral reflux, urinary tract infection, and nephrolithiasis. However, we have further encountered patients with more complex issues, such as chronic kidney disease (CKD), end-stage renal disease (ESRD), bilateral nephrectomies, and urinary diversion/augmentation surgeries. Of particular interest, there is a lack of standardized guidance for bladder management in SCI/D patients with ESRD. These patients are at high risk for urological complications and would benefit from codified bladder management strategies.

**Case presentation:**

In this article, we present eleven unique cases of NLUTD with associated ESRD and discuss recommendations utilizing simple and commonly available clinical interventions.

**Discussion:**

The inherently small population size of SCI/D patients with NLUTD and ESRD makes detailing a large sample size case series difficult. Future studies must aim to include a larger sample size as able, however, to better determine standardized protocols for chronic bladder management in SCI/D patients with NLUTD and ESRD. Experiences from this small case series are offered for consideration.

## Introduction

Bladder control requires careful coordination of the central, somatic, and autonomic nervous systems [1]. Disruptions in these systems, as seen in spinal cord injuries/disorders (SCI/D), cause bladder dysfunction known as neurogenic bladder or neurogenic lower urinary tract dysfunction (NLUTD). Mismanaged NLUTD in patients with SCI/D can lead to permanent urological complications, such as hydronephrosis, vesicoureteral reflux, recurrent urinary tract infections (UTI), stone formation, hydronephrosis and/or renal failure; additionally, it may induce autonomic dysreflexia, affect self-care/self-esteem, social engagement, and ultimately diminish quality of life [2–4].

Initial management of NLUTD is well documented, both in the acute care setting and during inpatient rehabilitation [5, 6]. Chronic management is variable, however, and depends on factors such as functional status, bladder type, and importantly on the presence of urogenital or renal complications. We have encountered patients with complex issues such as chronic kidney disease (CKD), end-stage renal disease (ESRD), bilateral nephrectomies, and urinary diversion/augmentation surgeries. Optimal management strategies for those with SCI/D and ESRD is of particular interest.

We reviewed published literature regarding standardized management of chronic NLUTD and ESRD. Search terms included spinal cord injury, bladder, renal failure, and NLUTD in several combinations. Minimal published information was found regarding standardized management of chronic NLUTD with associated renal failure. Most of reviewed literature documents outcomes (i.e., mortality) related to renal failure and UTIs, as well as the feasibility of hemodialysis utilization in the SCI/D population, but not on bladder management itself [7–9]. Optimal bladder management strategies in this subpopulation are unknown, warranting further evaluation.

## Methods

Our study personnel reviewed our local SCI/D patient registry at our local veteran’s health care system and identified eleven veterans with SCI/D, NLUTD, and ESRD (Table 1). The bladder management strategies employed for each individual patient are presented below. NLUTD was managed with simple and commonly available clinical interventions. Neurologic classification of patients adhered to the ISCoS International Standards for Neurological Classification of Spinal Cord Injury (ISNCSCI). Outcome results, where available, were reviewed retrospectively over a period of 10 years.

**Table 1:** Summary of demographics, SCI/D and medical conditions, complications, and management.

Case	Sex	Age	Classification	Etiology	Condition	Complications	Management
**1**	M	68	C4 AIS B	Transverse myelitis	NLUTD, ESRD with oliguria	Urethritis and recurrent UTIs. Deceased from respiratory failure secondary to intracranial hemorrhage	HD. Foley transitioned to daily bladder scan and IC if residual; 50–75 ml urine made daily
**2**	M	64	T4 AIS A	Motor vehicle accident	NLUTD, ESRD with anuria	UTI, urethritis	HD, bladder irrigation with Renacidin then normal saline weekly
**3**	F	65	C5 AIS D	Fall	NLUTD, RCC s/p bilateral nephrectomy; subsequent anuria	Urethritis, vulvovaginitis, recurrent UTIs	HD, bladder irrigation with normal saline until clear return weekly
**4**	M	73	C1 AIS D	Osteomyelitis	NLUTD, CKD4, BPH	Progression to ESRD with oliguria then anuria; UTIs. Deceased from cardiac arrest after aspiration	HD, IC q8h (then qhs, then no IC). Daily bladder irrigation for UTI prophylaxis
**5**	M	67	C4 AIS C	Fall	NLUTD, renal failure s/p renal transplant	Transplant failure and recurrent ESRD on HD, UTIs, trabeculations, urethral diverticula. Deceased, unknown cause.	HD, IC q6h volumes less than 400 ml daily, cystoscopy
**6**	M	81	C3 AIS C	Fall	NLUTD, ESRD with oliguria	Massive prostatomegaly, probable RCC, hematuria, non-obstructing nephrolithiasis. Deceased, unknown cause	HD. Monthly foley exchange due to prostatomegaly; 25 ml daily urine production, annual CT urogram
**7**	M	74	C4 AIS D	Cervical spondylotic myelopathy	NLUTD, ESRD with oliguria, BPH	Urosepsis from urinary stasis (NLUTD), oliguria. Deceased from unclear causes	HD. Initially with foley, then IC, then spontaneous voiding of at least 200 ml daily
**8**	M	69	C5 AIS D	Cervical spondylotic myelopathy	NLUTD, ESRD with anuria	Hematuria (?hemangiomas), urosepsis, drainage	Pads, adult garments as refused IC
**9**	M	58	C6 AIS D	Cervical spondylotic myelopathy	NLUTD, ESRD with oliguria, renal cyst	Voiding volitionally, condom catheter for convenience	Volitional void (at least 500 ml daily), HD
**10**	M	74	T10 AIS A	Motor vehicle accident	NLUTD, chronic pyelonephritis, renal stones, UTIs, ?RCC, hydrocelectomy, CKD	Non-oliguric AKI on CKD: new HD need. Declined community HD. Complicated UTI vs pyelonephritis	Left against medical advice. Continued Foley ( > 1000 ml daily urine production)
**11**	M	66	C1 AIS D	Fall	NLUTD, ESRD with oliguria	Recurrent UTIs, bacteremia	Chronic foley with monthly exchange, 700 ml daily, HD

### Case 1

A 68-year-old male with C4 AIS B tetraplegia secondary to transverse myelitis, chronic respiratory failure with ventilator dependence, ESRD with oliguria requiring hemodialysis (HD), hypertension, type 2 diabetes mellitus (DM2), coronary artery disease (CAD), dysphagia with chronic enteral nutrition, and chronic stage 4 sacral pressure injury was followed in clinic. He had a long-standing history of ESRD but still made 50–75 milliliters (ml) of urine daily. He historically was managed with an indwelling transurethral (Foley) catheter. He had experienced recurrent UTIs and urethritis despite optimized catheter care and frequent catheter exchanges. Prior urologic and renal evaluations including surveillance cystoscopy only revealed urethritis. Sexually transmitted diseases (STDs) were ruled out. We discontinued the Foley catheter and initiated daily bladder scanning and clean intermittent catheterization (IC) for residual. This was continued for 18 months, and he subsequently experienced fewer UTIs and no further episodes of urethritis. He later passed away from respiratory failure after intracranial hemorrhage post intracranial abscess evacuation.

### Case 2

A 64-year-old male with T4 AIS A paraplegia secondary to motor vehicle accident (MVA), ESRD with anuria requiring HD, DM2, chronic obstructive pulmonary disease (COPD), and bilateral chronic stage 4 ischial, sacral pressure injuries was admitted to the hospital. During his extended hospitalization for wound care, we noted urethral discharge and vague symptoms of UTI. He was treated on multiple occasions for possible cystitis based on cultures and the presence of urethral discharge. Other possible causes such as STDs were ruled out, but his condition remained. We initiated weekly bladder irrigation with citric acid/glucono-delta-lactone/magnesium carbonate (Renacidin) followed by normal saline (NS) as needed until there was clear return. We followed the results of this intervention for 3 months and noted far fewer episodes of urethral discharge and presumed UTI (2 episodes of minimal urethral discharge and 1 possible UTI as compared with 6 episodes of urethral discharge and 3 UTIs before intervention). Further outcomes are unknown.

### Case 3

A 65-year-old female with acute C5 AIS D tetraplegia secondary to a fall, renal cell carcinoma (RCC) status post bilateral nephrectomies on HD, breast cancer (status post right sided mastectomy with completion of chemotherapy), DM2, and hypertension was admitted for acute rehabilitation. Although she was anuric and required HD thrice weekly, she developed frequent vulvovaginal fungal infections with episodic urethral discharge and urethritis. STDs were ruled out. She had optimized glycemic control and was treated with antifungals on multiple occasions during her hospitalization with some improvement. We initiated weekly bladder irrigation with NS (no Renacidin due to vulvovaginitis) until there was clear return. She had two irrigations while inpatient and we recommended continuing irrigation once discharged. After 14 months of follow-up, results showed decreased frequency of vulvovaginitis and urethritis (2 episodes in 14 months compared with 5 episodes in the previous 17 months).

### Case 4

A 73-year-old male with chronic C1 AIS D tetraplegia from osteomyelitis/discitis in the setting of cervical fusion for myelopathy, heart failure with preserved ejection fraction (HFpEF), CKD4, type 1 DM with nephropathy, suspected benign prostatic hyperplasia (BPH), heart block status post pacemaker, seizures from prior intracranial hemorrhage, and history of varicella zoster encephalitis was seen on consults. The patient’s initial injury was several years prior; however, he was first seen by SCI/D after being admitted for volume overload from progression of CKD to ESRD requiring HD, when it was discovered that he had sustained a remote SCI. SCI interview noted likely neurogenic bowel and bladder (made some unknown amount of urine) that patient was managing with adult diapers. Formal bowel/bladder program and SCI rehab occurred. For NLUTD management he was started on IC three times daily. His course was complicated by UTIs. In the outpatient setting he was maintained on daily bladder irrigation for UTIs/pyocystitis suppression. His urine output declined to the point that bedtime catheterization was used and then ultimately no catheter usage was required due to anuria, though he continued daily bladder irrigation for prophylaxis. He ultimately passed after aspiration leading to cardiopulmonary arrest.

### Case 5

A 67-year-old male with history of ESRD status post renal transplant requiring HD, hypertension, CAD, cervical stenosis, who developed C4 AIS C tetraplegia after traumatic odontoid fracture, status post cervical fusion and laminoplasty, complicated by epidural hematoma post operatively leading to ascending paralysis and complicated ICU course including cardiac arrest, sepsis, and respiratory failure was evaluated. HD was reinitiated due to transplanted kidney failure. After stabilization, he was transferred for spinal cord rehab. He still made some urine and thus started IC every 4–6 h, with averages volumes of 150 ml. Over the next two-three years, he developed multi-drug resistant UTIs and hematuria. Cystoscopy on subsequent annual examination noted moderate hypertrophy of the lateral lobe of the prostate, very severe trabeculations, and urethral diverticula. Total daily urine production declined to 400 ml with IC q6 h. He continued to follow with urology for outpatient monitoring. He later passed away at home from unknown causes.

### Case 6

An 81-year-old male with history of ESRD with oliguria requiring HD, CAD, deep vein thrombosis, HFpEF, DM2 was admitted for rehabilitation of central cord syndrome after a hyperextension injury leading to C3 AIS C tetraplegia status post anterior corpectomy and posterior decompression, fusion complicated by respiratory failure and hospital acquired pneumonia. During the hospital course, CT urogram detected probable RCC, non-obstructing bilateral nephrolithiasis, and hematuria workup (cystoscopy) ultimately led to the detection of massive prostatomegaly. IC was attempted to management his residual urine production (less than 25 ml daily). However, given massive prostatomegaly he ultimately required an indwelling foley catheter. Ultimately, he discharged to a skilled nursing facility (SNF) with plans for monthly foley exchange and annual CT urogram to monitor suspected RCC. He was later admitted to a local medical center and passed away from unknown causes.

### Case 7

A 74-year-old male with ESRD with oliguria requiring HD, CAD on dual antiplatelet therapy, stroke with residual right hemiparesis, suspected BPH, DM2, and known cervical spinal stenosis with progressive spondylotic myelopathy was admitted to an outside hospital due to acute encephalopathy after an increase in his opioid regimen due to neck pain. He was transferred for neurosurgical evaluation once stabilized and underwent posterior laminectomy and fusion. His acute care course was notable for septic shock in the setting of a multi-organism UTI and aspiration pneumonia. In the ICU, it was noted that he was incontinent, and did have ongoing urine production but with new urinary retention, and initially had a Foley catheter placed. On SCI evaluation, he was determined to have C4 AIS D tetraplegia. Once stabilized, he was accepted into spinal cord rehabilitation, where daily IC was utilized given initial failure of voiding trials. His home tamsulosin was restarted. He eventually demonstrated spontaneous voiding (daily volumes near 200 ml) with minimal residual volumes, so IC was discontinued. He was discharged to the community with annual follow planned. He later passed away from unclear causes.

### Case 8

A 69-year-old male with a history of ESRD with anuria on HD, who developed C5 AIS D tetraplegia due to cervical spondylotic myelopathy, status post C3-C6 posterior spinal fusion was evaluated. He subsequently developed dark, bloody drainage when his postoperative foley was removed. He was evaluated by urology and a month later (with interval resolution of hematuria) underwent cystoscopy and ureteroscopy and was found to have subcentimeter hemangioma-like lesions throughout his bladder. Postprocedurally, he developed sepsis, requiring intravenous antibiotics. He continued to have urgency, intermittent hematuria, purulent discharge, and low-grade fevers. He refused IC due to pain. His leukocytosis improved, though he continued to have intermittent penile drainage, and was ultimately managed with pads and discharged home with plans for further outpatient follow up.

### Case 9

A 58-year-old with history of HTN, ESRD on HD with oliguria, Bosniak 3 left renal cyst, was admitted to outside hospital after C6-C7 spinous fracture after ground level fall, status post C5-T1 posterior spinal fusion, transferred to rehab for management of C6 AIS D tetraplegia. Based on history and evaluation he was found to be able to continue to void spontaneously, though typically utilized condom catheter for convenience. He typically voided more than 500 ml daily into collection devices. He continued spontaneous voiding into collection devices along with HD on discharge with plans for ongoing outpatient follow up.

### Case 10

A 74-year-old with history of chronic T10 AIS A paraplegia secondary to MVC, NLUTD with chronic foley use, renal mass (unclear if RCC, but status post radiofrequency ablation), left staghorn renal calculus status post percutaneous nephrolithotomy tubes with subsequent chronic left xanthogranulomatous pyelonephritis with worsening CKD followed by urology was evaluated. Of note, he had a recent outside hospital admission to the ICU for urgent HD and anemia treatment as well as left hydrocelectomy. He was readmitted to our institution with worsening leukocytosis in the setting of complicated UTI versus pyelonephritis and worsening of baseline renal function (non-oliguric AKI3 on CKD4) with recommendation from consulting teams for permacath placement, HD, and nephrectomy. While he initially allowed urgent HD, he declined surgical intervention and permacath placement for community HD. He continued to make over 1000 ml daily with Foley catheter in place. He ultimately left against medical advice, declining nephrectomy or plans for community HD, with tentative plans to follow up outpatient at local institutions near his home. Follow up with urology, SCI/D services was attempted post discharge, however contact was not able to be achieved.

### Case 11

A 66-year-old with HFrEF, ESRD on HD with oliguria, who sustained C1 AIS D tetraplegia related to a fall in the setting of encephalopathy after missing HD with underlying cervical spondylosis was evaluated. He ultimately required indwelling foley catheter given residual urine production as well as poor finger flexion, and was discharged to SNF after an acute inpatient rehab course. He subsequently had multiple hospital readmissions, including for bacteremia and UTI leading to septic shock and respiratory failure, ultimately thought to be related to cholecystitis with hematogenous spread. He initially refused surgical intervention for cholecystitis, but was eventually amenable with symptomatic improvement postoperatively. He later underwent formal urodynamic studies and was found to have a reduced bladder capacity and compliance. He elected to continue chronic foley use and continued to produced more than 700 ml of urine daily. He was discharged back to SNF where he continues to reside with annual outpatient evaluation anticipated.

## Discussion

The survival rate for SCI/D patients with ESRD is lower than the general population of ESRD patients, with chronic infection, urolithiasis, and secondary amyloidosis being common causes of death [8, 9]. In the series of eleven patients presented, at least five were deceased within 6 years of their initial injury; of note, more than one patient was lost to follow up so it is unclear if the mortality rate is actually higher. Of the five patients who were known to have died, one died from complications from an intracranial hemorrhage, one from complications from aspiration, and the other three from unknown causes with no autopsy report readily available.

Managing NLUTD in SCI/D with associated unique urologic and renal conditions is highly complex and challenging. Inadequate or inappropriate bladder management for these patients may put them at additional risk for additional sequelae which in turn may increase morbidity and mortality. This is particularly worrisome with the aging SCI/D population and the expectation that more patients with NLUTD and ESRD will present with complex care needs as discussed. As such, identifying common themes in presentation and associated sequelae are critical to inform management strategies.

A common theme across patients in this series was the importance of determining the patient’s baseline level of urine production (particularly if ESRD was present prior to SCI/D); several complications occurred due to patients with oliguria being assumed to have anuria due to acute NLUTD. Oliguria versus anuria is also important to differentiate as contrasted urologic studies may theoretically convert an oliguric patient into a completely anuric patient (i.e., due to a contrast induced nephropathy) [10]. Outside the genitourinary and renal systems, the effects of SCI/D, NLUTD, and ESRD continue to be explored; small case series have noted, for example, gastrointestinal complications such as amyloidosis, gallbladder pathology, gastric disease, and bowel complications in those with SCI/D with ESRD on HD, though causality has not been established in robust, comparative studies [11].

## Conclusions

Currently, there is no standard bladder management protocol or algorithm for this subpopulation to minimize aforementioned risks; however, our small case series is an initial attempt to highlight factors to consider in any potential future guidelines. Large scale studies are difficult given the inherently small subpopulation with SCI, NLUTD, and ESRD, as well as the variability in extent, timing, and type of interrelated pathologies. Even so, it is worth pursuing future larger scale studies to inform the creation of a standardized protocol or algorithm for bladder management in this cohort. A database or registry of these patients would be of particular benefit to increase the power of future studies. Consequently, we anticipate collecting and publishing a larger case series for further evaluation, with an overarching goal of contributing to uniform guidelines for managing NLUTD in patients with SCI/D and ESRD.
